# Large EEG amplitude effects are highly similar across Necker cube, smiley, and abstract stimuli

**DOI:** 10.1371/journal.pone.0232928

**Published:** 2020-05-20

**Authors:** Ellen Joos, Anne Giersch, Lukas Hecker, Julia Schipp, Sven P. Heinrich, Ludger Tebartz van Elst, Jürgen Kornmeier

**Affiliations:** 1 INSERM U1114, Cognitive Neuropsychology and Pathophysiology of Schizophrenia, University of Strasbourg, Strasbourg, France; 2 Department of Psychiatry and Psychotherapy, Medical Center—University of Freiburg, Faculty of Medicine, University of Freiburg, Freiburg, Germany; 3 Institute for Frontier Areas of Psychology and Mental Health Freiburg, Germany, Germany; 4 Section for Functional Vision Research, Eye Center, Medical Center—University of Freiburg, Faculty of Medicine, University of Freiburg, Freiburg, Germany; Psychologische Hochschule Berlin, GERMANY

## Abstract

The information available through our senses is noisy, incomplete, and ambiguous. Our perceptual systems have to resolve this ambiguity to construct stable and reliable percepts. Previous EEG studies found large amplitude differences in two event-related potential (ERP) components 200 and 400 ms after stimulus onset when comparing ambiguous with disambiguated visual information ("*ERP Ambiguity Effects*"). These effects so far generalized across classical ambiguous figures from different visual categories at lower (geometry, motion) and intermediate (Gestalt perception) levels. The present study aimed to examine whether these ERP Effects are restricted to ambiguous figures or whether they also occur for different degrees of visibility. Smiley faces with low and high visibility of emotional expressions, as well as abstract figures with low and high visibility of a target curvature were presented. We thus compared ambiguity effects in geometric cube stimuli with visibility in emotional faces, and with visibility in abstract figures. ERP Effects were replicated for the geometric stimuli and very similar ERP Effects were found for stimuli with emotional face expressions but also for abstract figures. Conclusively, the ERP amplitude effects generalize across fundamentally different stimulus categories and show highly similar effects for different degrees of stimulus ambiguity and stimulus visibility. We postulate the existence of a high-level/meta-perceptual evaluation instance, beyond sensory details, that estimates the certainty of a perceptual decision. The ERP Effects may reflect differences in evaluation results.

## Introduction

The information available through our senses is incomplete, noisy and sometimes ambiguous. For example, we see objects only from one perspective, they are often partially occluded, or seen in suboptimal conditions e.g. during rain or fog. Further, the available sensory information at a given moment can be ambiguous and thus allow for several about equally probable but mutually exclusive interpretations. The perceptual system has to overcome such sensory limitations and resolve ambiguities in order to create stable and reliable representations of the external world [1].

**Fig 1:**
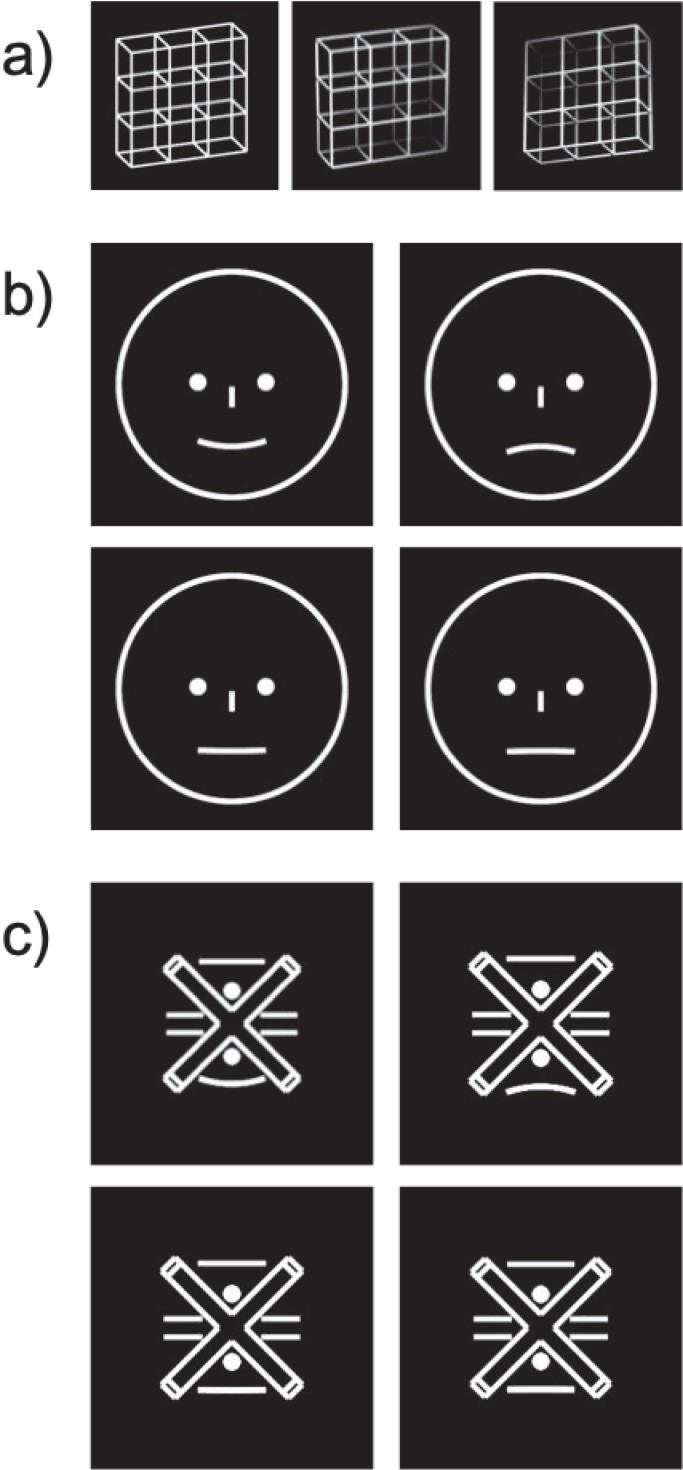
Stimuli. (a) depicts the ambiguous Necker lattice (left) and the disambiguated variants thereof (middle and right). Smiley (b) and abstract figure (c) stimuli are enlarged for better visibility of the "mouth" curvature. (b) depicts the high-visibility (upper row) and low-visibility (bottom row) smileys. Happy smileys are depicted in the left column, sad smileys in the right column. The emotional expression of the smileys was only created through the mouth curvature. In a control condition we embedded the same mouth curvatures into abstract figures (c). Strongly bended “mouth” curvatures are depicted in the upper row and slightly bended “mouth” curvatures in the bottom row (upwards on the left, downwards on the right).

Ambiguity can arise in different modalities [2], in different forms [3,4], and at different levels of stimulus complexity. Most prominent scientific examples of ambiguity are classical ambiguous figures like the Necker cube [5] (see the Necker lattice [6,7], a variant of the Necker cube in Fig 1A left graph) or Rubin's face-vase illusion [8]. Here one and the same sensory information allows for two or more possible and about equally probable interpretations. During prolonged observation, our perception becomes unstable and alternates repeatedly between different interpretations [see 3 for a review of the phenomenon].

Interestingly, there are many different uses of the term "ambiguity". One prominent area beyond perception science is art, where–at first sight–this term seems to have a different meaning. One most prominent example is Da Vinci's famous "Mona Lisa" painting. The English essayist and writer Walter Pater affirmed in a prominent essay, that Mona Lisa's smile holds an "emotional ambiguity", revealing first a "promise of an unbounded tenderness", but soon after also a "sinister menace" [9]. A large number of articles about Mona Lisa focus on this ambiguity in her emotional facial expression [10–12], which seems to be very different from the ambiguity examples in perceptual science. Mona Lisa's emotional expression is not perceived as either clearly happy or clearly sad but rather more or less happy or sad. Several nuances of emotional expressions and therefore several slightly different interpretations are theoretically possible when observing Mona Lisa.

There is an obvious qualitative difference in the perception between such types of ambiguity and ambiguity as used with the classical ambiguous figures, like the Necker cube. In the case of the Necker cube, perception oscillates between two clear-cut perceptual alternatives, i.e. a perspective from above and a perspective from below. Therefore the perceptual decision here seems to be binary. The interpretation of emotional facial expressions, on the other hand, has different preconditions and is probably more complicated compared to the Necker cube. The relations between the relevant face muscles [13] need to be analysed and related to emotional states experienced by ourselves. The necessary reference system for emotional states is thus endogenous and theory of mind concepts are needed [see 14 for related concepts of embodiment]. Based on internal perceptual statistics generated from memorized perceptual experiences over lifetime, specific patterns of face-muscle-relations receive specific probability values for representing certain emotional states. The ambiguity of an emotional face is thus rather based on a continuous scale of theoretically possible perceptual outcomes.

**Fig 2:**
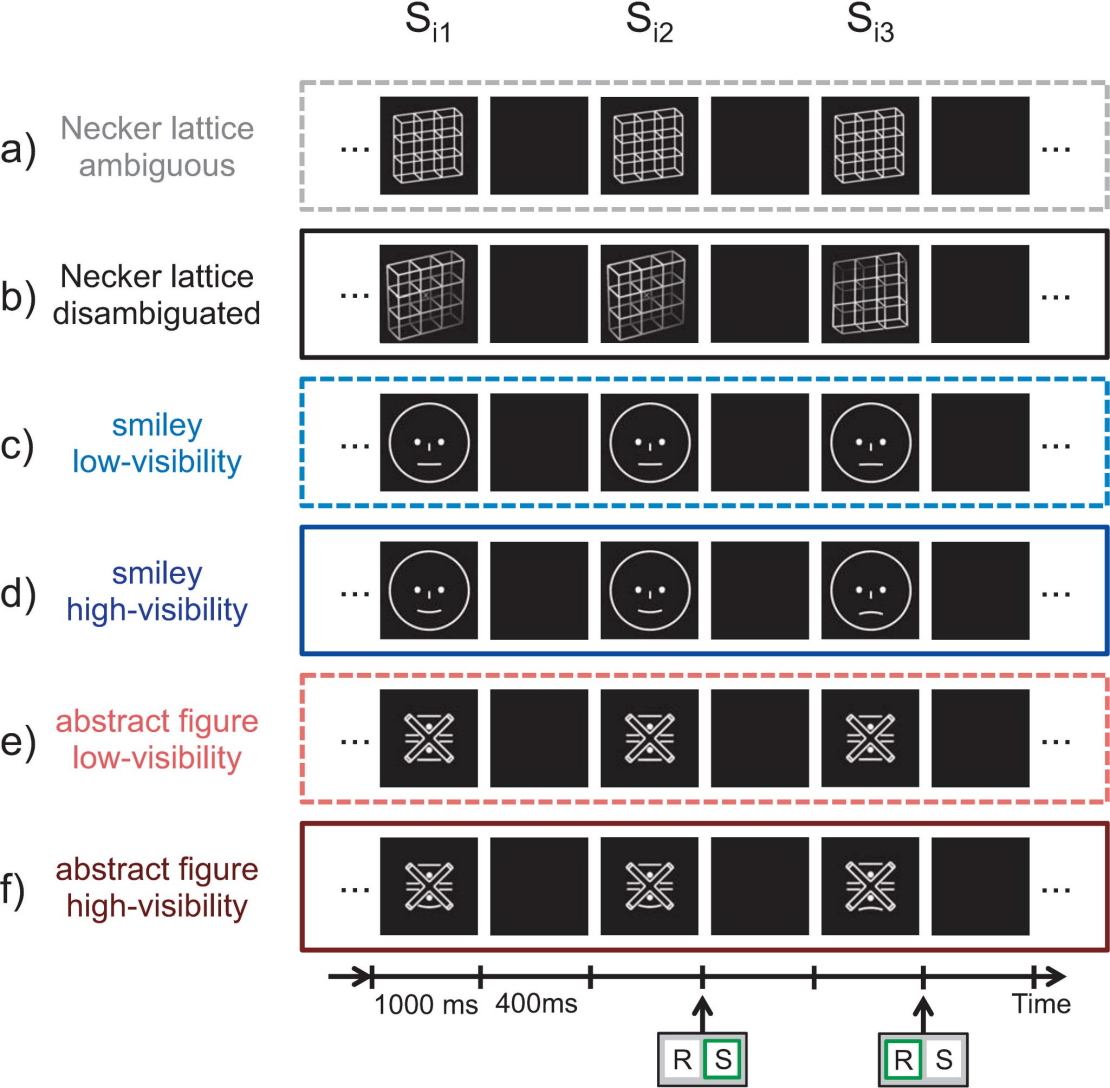
Experimental paradigm. Stimuli were presented discontinuously for 1000 ms with an inter-stimulus interval of 400 ms. Participants compared the current stimulus with the immediately preceding one. In case of identical percepts across two consecutive stimuli, participants pressed 'S' (stability) on a keyboard (represented as squares with the letters 'R' and 'S' below the time axis). If perception changed from one stimulus to the next, participants pressed 'R' (reversal). Stimulus type and ambiguity/visibility level stayed unchanged within experimental conditions (within rows), but differed between experimental conditions (different rows). The order of conditions (rows) was pseudo-randomized. The specific sequences of stimuli (Si1, Si2, Si3) in b—f are for demonstration purposes.

A closer look at the Necker cube relativizes its binary nature. The source of ambiguity of the Necker cube is the projection of a 3D world on 2D retinae during the first step of vision, and the fact that two different 3D grid objects produce identical projections on the retinae. However, one could imagine in principle infinitively many other 3D grid objects and even some 2D objects projecting identically on the retinae as the Necker cube, as Fig 2C in Kersten and Yuille [15] demonstrates nicely. This implies that the theoretically possible interpretations of the Necker cube are manifold rather than twofold. The reason for the preference of two 90° object interpretations is simply that 90° angles are much more frequent in our environment than any other angles and thus more probable. To reduce the infinitely many possible interpretations of the Necker cube to the two most probable interpretations, one "only" has to match the sensory evidence to an internal statistics about perceptual experiences, learned over lifetime.

Thus, ambiguity of emotional faces and of geometric cube stimuli may share one basic principle. The fact that the identical sensory information is compatible with several theoretically possible perceptual interpretations holds for both, the Necker cube and Mona Lisa, and reflects the ambiguity of both stimulus types. However, the reduction of the number of possible interpretations to a lower number of highly probable interpretations, the underlying probability distributions, and the subjective experience seem to differ. The commonality between Necker cube perception and perception of "ambiguous" emotional face expressions may thus be the resulting perceptual uncertainty during their observation, given insufficient sensory evidence.

Interesting in this context are two EEG studies by Kornmeier et al. [16,17]. They compared event-related potentials (ERPs) evoked by ambiguous and disambiguated versions of the Necker cube and found unusually large amplitude differences in two components, an anteriorly distributed P200, followed by a posteriorly distributed P400. Both components show small amplitudes for ambiguous stimuli and large amplitudes for the respective disambiguated stimulus variants. The same pattern of results was found for ambiguity in motion (von Schiller's stroboscopic alternative motion stimulus—also known as the SAM/'motion quartet' [18]) and also in Gestalt perception (Borings ambiguous Old/Young Woman [19]). This is remarkable, because of the dramatic differences in the low-level features and in the sources of ambiguity. One obvious commonality between these different stimulus types is ambiguity at a more abstract level, i.e. that one and the same sensory information is about equally compatible with different interpretations. Based on this consideration Kornmeier et al. labelled these effects the "ERP Ambiguity Effects" [16,17] with ambiguity representation at a higher-level, beyond sensory details. In the current study we were interested in whether the “ERP Ambiguity Effects” further generalize across stimuli with emotional facial expressions and thus whether they may be rather "ERP Uncertainty Effects". Until this issue is resolved, we decided to adopt a neutral nomenclature, “ERP Effects”, for the methods, results and part of the discussion section.

The above-mentioned example for "ambiguity" in emotion, the Mona Lisa, is a highly complex painting, which has multiple sources for uncertainty. We aimed at having maximal control over the source of uncertainty and thus created simpler line drawings of a face (smileys). It has been found, that the mouth region is of high importance for emotion perception in faces [e.g. 20], therefore we only varied the mouth curvature of the smileys to introduce happy and sad emotional facial expressions. We created two smiley variants with highly visible happy and sad expressions (mouth curvatures with strong bending), corresponding to the disambiguated versions in the Necker lattice. Both stimulus types (smiley, Necker lattice) should result in perceptual outcomes with low uncertainty. We further created two smiley versions with less visible emotional expressions (mouth curvatures with weak bending) that could be perceived either as slightly happy or as slightly sad, corresponding to the ambiguous Necker lattices and evoking high perceptual uncertainty. The choice of specific smiley stimulus variants was based on a pilot psychophysical experiment (for details see Supporting Information S1 File). There, participants were instructed to make binary decisions concerning the perceived emotional expression (happy/sad) of the smiley faces. With these responses we could identify those stimulus variants that were perceived in half of the trials as happy and in the other half as sad (low-visibility), which were chosen as the “less visible” emotion expressing stimulus variants. We here define the term "visibility" as the ability to spatially resolve the difference between the mouth curvatures bending.

The smiley stimuli evoke perceptual (un)certainty due to the visibility of their mouth curvatures. These, in turn, evoke the perception of emotional expressions (see e.g. Fig 1B). Emotional expressions are inevitably linked to their low-level features (e.g. mouth curvature) and cannot be studied in isolation. This makes assumptions about the origin (line bending or emotional expression) of perceptual (un)certainty in the case of smileys difficult in the current study.

Face processing, however, is known to be holistic in the sense that the individual stimulus features are processed and integrated simultaneously rather than in a hierarchical manner [21,22]. Thus, if the smileys are perceived as faces they should also be processed holistically. Then the mouth curvature should be integrated into the face, automatically resulting in the percept of an emotional face. To test whether smileys were at all perceived as faces, we introduced a control condition with "abstract figures" containing the same low-level stimulus features as in the smileys. However, these low-level visual details were differently arranged to prevent the recognition of a face, with one exception: the curvatures representing the mouth in the smiley stimuli were presented in the same size and at the same position within those abstract figures as in the smileys. This abstract figure condition had two purposes: (1) to investigate if face-specific ERP signatures are present with smileys and absent with abstract figures. (2) Both the occurrence of the ERP Effects in very different stimulus types (Necker cube, SAM, Boring's Old/Young Woman) and their relatively late occurrences (on a visual processing time scale) indicate that the ERP Effects reflect higher-level processes beyond stimulus specific features. Therefore, the second purpose of presenting the abstract figures was to investigate whether the ERP Effects also generalize across the curvature ambiguity in abstract figures.

## Methods

### Participants

Twenty healthy participants (11 females) between 19 and 34 years (mean: 25.1 years) with normal or corrected-to-normal visual acuity participated in this study. All gave their informed written consent. The study was approved by the ethics committee of the University of Freiburg and performed in accordance with the ethical standards laid down in the Declaration of Helsinki [23].

### Stimuli

Three stimulus types were used: Necker lattice stimuli, smileys, and abstract figures (see Fig 1). In separate experimental conditions, either ambiguous or disambiguated variants of the Necker lattice were presented. Smileys could have either low or high visibility of the mouth curvature. The respective separate experimental conditions are labelled as "low-visibility smileys" and "high-visibility smileys". Abstract figures could have either low or high visibility of a line curvature located beneath the fixation cross. The respective separate experimental conditions are labelled as "low-visibility abstract figures" and "high-visibility abstract figures".

The ambiguous Necker lattices–a combination of nine Necker cubes (Fig 1A left graph, [5,7])–and disambiguated Necker lattices were presented in white on a dark background. The stimuli had a size of 7.5°**×**7.5° degrees of visual angle ("VA"). We created two disambiguated Necker lattice variants corresponding to the two perceptual interpretations of the ambiguous Necker lattice by adding depth cues like shading, central projection, and aerial perspective [see 24 for the OpenGL lighting model]. Both ambiguous and disambiguated Necker lattice variants had an overall luminance of 40 cd/m^2^. For the disambiguated Necker lattice variants this luminance value represents an average across corners. A cross in the centre of the Necker lattices served as fixation target.

The present smileys were emotional face stimuli (see Fig 1B) with a minimal parameter space that allows maximal stimulus control and thus makes it easy to quantify levels of low and high visibility of the emotional expression. The face border was described by a white circle with a diameter of d = 4° VA on a black background. The eyes were two filled circles with a diameter of 0.214° VA and a distance to the face symmetry axis of 0.611° VA to the left and right respectively. The nose was indicated by a simple vertical line with 0.377° VA length and 0.102° VA width, located on the face symmetry axis at 2.076° VA distance from the upper central face border. Two smiley variants with happy and sad expressions at two visibility levels with less and highly visible happy and sad expressions were produced. Happiness/Sadness was only controlled via the mouth curvature. The upper (sad expression) and lower (happy expressions) arcs of a circle with two different radii r (r = 100.662/4.601° VA for slightly/strongly happy and sad smileys) indicated the mouth.

In a pilot study those mouth curvatures were determined that could still be discriminated, but were as similar as possible. Therefore we used the method of constant stimuli [25] (see Supporting Information S1 File). The common anchor point of the four mouth variants/circle arcs was the central point of the circle arcs that was kept constant at a distance of 0.916° VA to the lower central face border across stimulus variants. Imaginary vertical lines at 0.611° VA left and right from the (vertical) face symmetry axis defined left and right end points of the four mouth variants/circle arcs.

In this study we created new smiley stimuli and thus had to verify that they were perceived as faces. Therefore, we introduced a control condition with the following stimuli: we used the same less and highly visible mouth elements/circle arcs as in the smileys and embedded them into an abstract figure. These abstract figures had the same total line length and luminance (40 cd/m^2^) as the smileys, but the line elements were arranged in a way that hardly any face could be recognized (see Fig 1C). Larger amplitudes of the face-specific N170 ERP component [26–28] for smileys compared to abstract figures would be evidence for face-specific processing of the smileys.

### Procedure

In total we presented six separate experimental conditions, with two ambiguity/visibility levels for each stimulus type (Necker lattice, smiley, abstract figure). In two experimental conditions, either ambiguous or disambiguated variants of the Necker lattice were presented. In two conditions either low-visibility or high-visibility smileys and in two other conditions either low-visibility or high-visibility abstract figures were presented.

Necker lattice blocks lasted for 7 minutes, smiley and abstract figures blocks lasted for 6 minutes. The order of experimental conditions within one day was pseudo-randomized. The measurements were performed within two sessions on two different days (median time between two sessions: 2 days, range: 1–6 days). The abstract figures and Necker lattices were always presented in the first session, the smileys in the second.

Within the disambiguated/high-visibility stimulus conditions, the two respective stimulus variants were alternated randomly to simulate the spontaneous perceptual reversals of the ambiguous variants. The disambiguated Necker lattice variants were alternated with a reversal probability of 30% according to the average reversal probability of the ambiguous lattices as known from the literature [29,30]. Low-visibility and high-visibility smileys and abstract figures were also presented with a 30% reversal probability.

Stimuli were presented discontinuously for 1000 ms with a blank inter-stimulus interval of 400 ms (see Fig 2 and [16,17]). Participants were instructed to compare their current percept to the immediately preceding percept and to indicate perceptual reversals (change from one percept to the other) or perceptual stability (identical percepts across two consecutive presentations) for each stimulus (Fig 2) by pressing different keys (‘S’ for stability and ‘R’ for reversals) on a keyboard with four keys (two keys were not used). Keys were pressed using the thumb and ‘S’ and ‘R’ assignment to the left or the right thumb was counterbalanced between participants. Further, participants were instructed to respond as quickly and as precisely as possible. For Necker lattices, the two possible percepts were front-side pointing upwards or downwards (Fig 1A middle and right graph). Smileys could be perceived as either happy or sad. For the abstract figures the ends of the bended line could be perceived as pointing either upwards or downwards.

### EEG recording and pre-processing

EEG was recorded with 32 active silver/silver chloride electrodes at scalp locations according to the extended 10–10 system [31]. Impedance was kept below 10 kΩ across electrodes. EEG data were digitized with 1000 Hz sampling rate, and online band-pass filtered with 0.01–120 Hz. Data analysis was executed in Igor Pro 6.3 (Wavemetrics, Inc.). The data was band-pass filtered offline at 0.01–25 Hz. It was re-referenced to the averaged mastoid channels for the analyses of P200 and P400 ERP Effects [16,17] and re-referenced to common average for the analyses of the face-specific N170 ERP component [26].

Trials exceeding an artefact threshold of ±100 μV were excluded from analysis. The baseline was defined as the average from 60 ms before to 40 ms after stimulus onset. For each stimulus type and ambiguity/visibility level, the EEG data was averaged separately for each participant and electrode. The trials started 60 ms before stimulus onset and were analysed until 1000 ms after onset.

### Behavioural analysis

For each stimulus type and ambiguity/visibility level, we analysed the median reaction times and interquartile ranges with Wilcoxon signed rank tests. Reaction times are defined as the time between stimulus onset and the key press. Responses were regarded as physiologically plausible when their earliest occurrence was 150 ms after stimulus onset and responses were regarded as valid until the end of the inter-stimulus interval (1200 ms after stimulus onset).

### ERP analysis

The analysis focused on the known ERP Effects consisting of two positive ERP components, a P200 with a latency of about 200 ms after stimulus onset and a fronto-central scalp distribution and a P400 with a centro-parietal scalp distribution [16,17] occurring 400 ms after stimulus onset. Following Kornmeier et al. [16,17], we focused on electrode Cz as spatial region of interest (ROI) for both ERP components and on temporal ROIs from 100 to 300 ms for the P200 and from 300 to 600 ms for the P400. We re-referenced the data to the mastoid electrodes P7 and P8.

We further analysed the N170, a negative ERP component 170 ms after stimulus onset most prominent at the temporal electrode positions, which is known from the face processing literature [26,32]. Spatial ROI for the N170 were electrodes P7 and P8, the temporal ROI was from 150 to 220 ms after stimulus onset [26]. For this analysis the data was re-referenced to common-average due to the spatial distribution of the N170 ERP component.

We identified the individual peak amplitudes in the respective spatial and temporal ROIs and measured the average voltage in a ±30 ms time window around the peak [33].

19 participants with at least 30 valid trials per condition were included in the statistical analysis (1 participant had less trials in one condition and therefore was excluded from the analysis). Due to low numbers of perceptual reversals, the statistical analyses were based only on stability trials (see further elaboration in the results and discussion sections).

We conducted separate repeated-measures ANOVAs (rmANOVA) in SPSS (Version 24.0) with the variable *amplitude* for the P200 and the P400 ERP components, both with the factors *stimulus* (Necker lattice, smiley, abstract figure) and *sensory evidence* (ambiguous/low-visibility, disambiguated/high-visibility). A separate rmANOVA was conducted for the N170 component for the variable *amplitude* with the factors *stimulus* (smiley, abstract figure), *sensory evidence* (ambiguous/low-visibility, disambiguated/high-visibility) and *channel* (P7, P8). In case sphericity was violated the respective degrees of freedom and p-values were Greenhouse-Geisser corrected [34].

*P*-values resulting from the rmANOVAs, post-hoc *t*-tests and reaction time analyses were corrected for multiple testing using the Bonferroni-Holm correction with an alpha of 0.05 [35].

All data (behavioural and EEG) of disambiguated/high-visibility stimulus variants represent only correctly identified stability trials. Because there are no correct answers in the case of ambiguous Necker lattices, all valid indications of perceptual stability trials were included. We adopted the same strategy for low-visibility smiley and abstract figure conditions.

## Results

### Behavioural results

#### Trial numbers

Participants responded to reversal and stability trials by using two different keys. In the case of disambiguated Necker lattices, high-visibility smileys, and high-visibility abstract figures participants responded correctly to stability trials in more than 97% (valid trials) of all stimulus presentations on average (disambiguated Necker lattices: Median 97.44%–IQR: 95.6–98.7%, high-visibility smileys: Mdn 100%–IQR: 98.68–100%, high-visibility abstract figures: Mdn 98.46%–IQR: 97.02–99.48%). Ergo less than 3% of all stability trials contained incorrect responses, non-responses or multiple responses to one stimulus presentation. They further responded correctly to reversal trials in more than 85% of all trials on average (disambiguated lattices: Mdn 90.77%–IQR: 89.37–93.51%, high-visibility smileys: Mdn 92.05%–IQR: 87.34–95.69%, high-visibility abstract figures: Mdn 85.37%–IQR: 77.42–93.5%). Ergo less than 15% of all reversal trials contained incorrect responses, non-responses or multiple responses to one stimulus presentation. These incorrect responses, non-responses and multiple responses to one stimulus presentation in disambiguated/high-visibility conditions were excluded from further analysis from both, stability and reversal trials.

In the case of ambiguous Necker lattices there were no “correct” responses, because one and the same stimulus variants was presented and only the perceptual responses were available. We analysed low-visibility smileys and low-visibility abstract figures according to the ambiguous Necker lattices and thus we did not separately analyse correct and incorrect responses to the physical stability and reversal trials, but only classified trials as valid or invalid perceptual responses.

Valid perceptual response trials in the case of ambiguous/low-visibility stimuli are trials where participants gave one response per stimulus in a predefined time-window (not before 150 ms and not after 1200 ms after stimulus onset), for which they had to press one of two predefined keys (indicating a stability or a reversal trial). Participants gave valid perceptual responses in more than 95% of all stimulus presentations on average (ambiguous Necker lattices: Mdn 96.16%–IQR: 95.4–98.33%, low-visibility smileys: Mdn 98.74%–IQR: 95.65–100%, low-visibility abstract figures: Mdn 95.86%–IQR: 91.11–100%). Ergo less than 5% of all trials contained invalid perceptual responses. The invalid perceptual responses in ambiguous/low-visibility conditions were excluded from further analysis.

Table 1 shows the remaining stability and reversal trials with and without EEG artefact removal due to body and eye movements, eye blinks, low-conductance electrodes, etc. One has to differ between perceptual reversal trials (endogenously determined) and physical reversal trials (exogenously determined by the stimulus program). For disambiguated/high-visibility conditions we included only correct responses to physical reversal trials. For ambiguous/low-visibility conditions we included all perceptual reversal trials, irrespective of the correctness regarding physical reversal trials (available for smileys and abstract figures, but not for Necker lattices). The resulting reversal rates can be seen in Table 1 column six, along with the physical reversal rate in column seven (30% for disambiguated Necker lattices, low- and high-visibility smileys, and low- and high-visibility abstract figures).

**Table 1:** Trial numbers.

	Number of reversal responses (incl. artefact trials)	Number of reversal responses (excl. artefact trials	Number of stability responses (incl. artefact trials)	Number of stability responses (excl. artefact trials)	Reversal rate (incl. artefact trials)	Physical reversal rate (percentage)
Necker lattice – disambiguated	102.58 ±36.09	149.74 ±16.35	254.63 ±78.94	391.53 ±18.58	27.27% (25.84–29.58%)	30%
Necker lattice – ambiguous	64.53 ±47.2	100.11 ±69.54	293.74 ±103.73	467.53 ±71.12	18.56% (8.62–25.41%)	N/A
Smileys – high-visibility	94.16 ±32.01	131 ±25.25	231.89 ±66.32	340.74 ±12.68	28.03% (24.64–31.2%)	30%
Smileys – low-visibility	29.79 (±32.69)	36.74 (±36.92)	317.16 (±109.08)	452.79 (±38.88)	6.18% (2.24–9.21%)	30%
Abstract figures – high-visibility	77.21 (±30.48)	118.11 (±21.78)	199.58 (±82.45)	323.74 (±25.06)	25.74% (23.13–28.68%)	30%
Abstract figures – low-visibility	20.42 (±22.76)	28.74 (±27.76)	280.89 (±114.24)	455.68 (±33.24)	5.5% (0.6–9.52%)	30%

Table 1 displays the mean number of reversal trials (±Standard deviation) excluding artefact trials across participants in the second column and the mean number of all valid reversal response trials including artefact trials (±SD) in the third column, separately for the experimental conditions (rows). Similarly for stability trials, trials excluding artificial trials are presented in column four and trials including artificial trials are presented in column five. Column six shows the median reversal rate in percentage (IQR) including artificial trials. Column seven shows the physical reversal rate implemented in the stimulus presentation program.

For low-visibility stimuli the perceptual reversal rate (smileys: 6.18%, abstract figures: 5.5%) is obviously different from the physically determined reversal rate (both 30%). Therefore, we did analyse the correct responses in the low-visibility conditions and found correct responses to low-visibility smileys in 9.86% (Median, IQR: 3.43–16.73%) and to low-visibility abstract figures in 4.88% (Median, IQR: 0.88–14.57%). This explains the large difference between physically determined and perceptual reversal rates and is most probably related to the low visibility of the relevant stimulus information (curvature).

The number of reversal trials for disambiguated/high-visibility conditions after artefact removal (Table 1 column 2) would be sufficient for an analysis of reversal trials. The number of reversal trials for ambiguous/low-visibility conditions after artefact removal (Table 1 column 2), however, is very low and even the mean value of low-visibility smileys and abstract figures is below the criterion of at least 30 valid trials per participant and condition.

Recent studies about the currently investigated ERP amplitude effects had fewer conditions and therewith more trials per condition. Separate analyses for stability and reversal trials were possible and thus realized in these studies [16,17]. The main difference between stability and reversal trials was an additional P3b component superimposed on the P400 in the reversal conditions. In the present study we were not interested in a surprise P3b component and thus restricted our focus on what had been labelled as the “ERP Ambiguity Effect”, which was also found for the stability trials. We thus decided to add another experimental condition instead of attempting to collect enough reversal trials.

#### Reaction times

**Fig 3:**
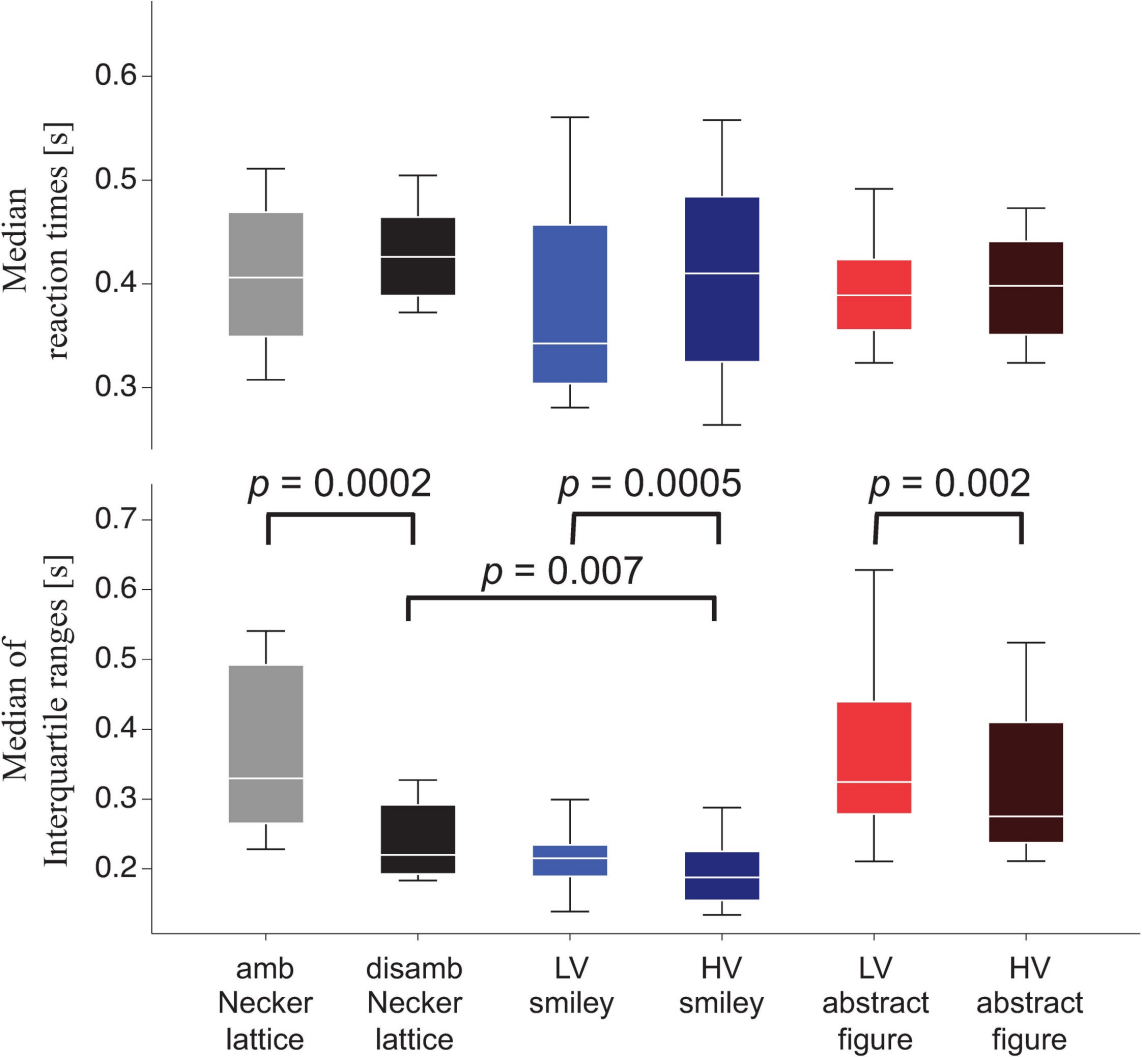
Reaction times. The upper row depicts the median (white line) of the individual median RTs (whiskers depict the interquartile range of reaction times). The bottom row depicts the intra-individual interquartile ranges of reaction times above (white line—median interquartile range; whiskers interquartile range of individual interquartile ranges; amb = ambiguous, disamb = disambiguated, LV = low-visibility, HV = high-visibility).

The Wilcoxon signed rank tests on median reaction times indicated no significant effects, neither for *sensory evidence* nor for *stimulus type* (see Fig 3 top row for graphical illustration and Supporting Information S1 Table).

For all stimulus types, the intra-individual interquartile ranges of the reaction times significantly differed between ambiguous/low-visibility and disambiguated/high-visibility stimulus variants (Necker lattices: *Z* = 3.78, *r* = 0.61, *p* = 0.0002; smileys: *Z* = 3.7, *r* = 0.6, *p* = 0.0005; abstract figures: *Z* = 3.54, *r* = 0.57, *p* = 0.002, see Fig 3 bottom row).

We further compared for each ambiguity/visibility level the intra-individual interquartile ranges of the reaction times between stimulus types. The Wilcoxon tests indicated a significant difference only between disambiguated Necker lattices and high-visibility smileys (*Z* = 3.3, *r* = 0.54, *p* = 0.007, see Fig 3 bottom row and Supporting Information S1 Table). In summary, we found equal median reaction times across stimulus types, but more reaction time variability for ambiguous/low-visibility compared to disambiguated/high-visibility stimulus variants.

### P200 and P400 ERP effects

One aim of this study was to replicate the P200 and P400 ERP Effects in the Necker lattice stimuli reported in previous studies [16,17]. A second aim was to investigate whether the effects are also present with the smiley stimuli and in the control condition with abstract figures.

**Fig 4:**
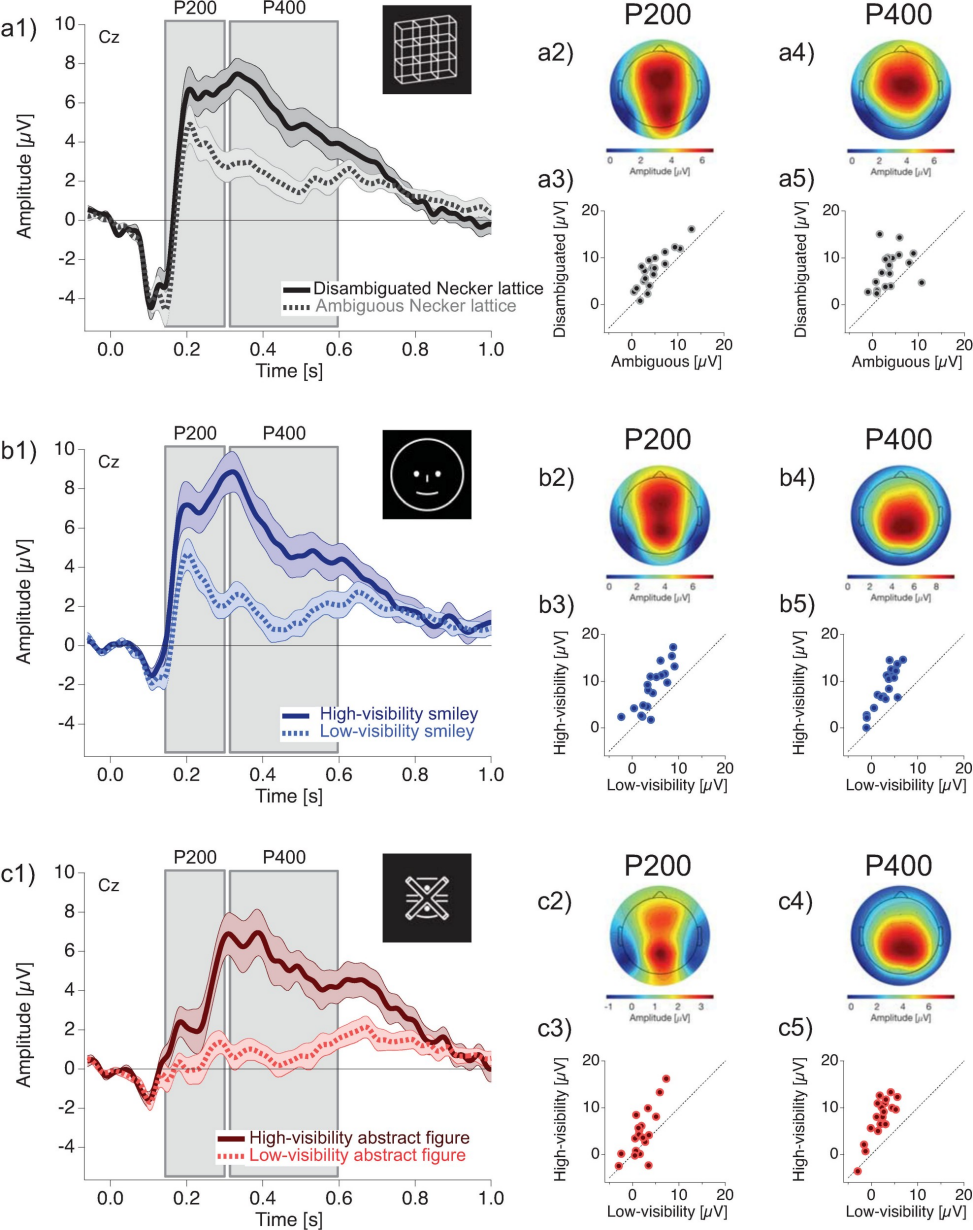
P200 and P400 ERP effects. P200 and P400 ERP Effects (re-referenced to the averaged mastoid electrodes) for Necker lattices (a1-a5), smileys (b1-b5), and abstract figures (c1-c5). Graphs (a1), (b1), and (c1) depict grand mean ERP traces for disambiguated/high-visibility (solid lines, dark colours) and ambiguous/low-visibility (dotted lines, light colours) stimuli. Graphs (a2, a4), (b2, b4), and (c2, c4) show grand mean voltage maps of the P200 (a2, b2, c2) and the P400 (a4, b4, c4) of the respective stimuli. Graphs (a3, a5), (b3, b5), and (c3, c5) show scatter plots for the P200 (a3, b3, c3) and the P400 (a5, b5, c5) with amplitudes of individual participants for the disambiguated/high-visibility (ordinate) versus ambiguous/low-visibility stimuli (abscissa). In all scatter plots the vast majority of data points are above the bisection line, indicating larger amplitudes for disambiguated/high-visibility compared to ambiguous/low-visibility stimulus variants.

Fig 4 (a1, b1, c1) displays the grand mean ERP traces at electrode Cz for disambiguated/high-visibility (solid lines) and ambiguous/low-visibility (dotted lines) stimulus variants. The P200 and the P400 show larger amplitudes with disambiguated/high-visibility compared to ambiguous/low-visibility stimuli.

The rmANOVA for the P200 ERP amplitudes showed a significant main effect of *sensory evidence* (*F*(1,18) = 37.6, *p* = 0.0002, $\eta_{p}^{2}$ = 0.68). The P200 has its maximal amplitudes from frontal to parietal electrodes near the midline for Necker lattices (Fig 4 a2), smileys (Fig 4 b2), and abstract figures (Fig 4 c2). Fig 4 a3, b3, and c3 depict scatterplots with P200 amplitudes from individual participants. Amplitudes are larger for disambiguated/high-visibility than for ambiguous/low-visibility stimuli (i.e. above the identity line) for the vast majority of participants (Necker lattices: 17 out of 19; smileys: 18 out of 19; abstract figures: 15 out of 19).

The rmANOVA for the P400 ERP amplitudes also showed a significant main effect of *sensory evidence* (*F*(1,18) = 27.15, *p* = 0.0013, $\eta_{p}^{2}$ = 0.6). The P400 has its highest activation at centro-parietal electrodes for all of the three stimulus types (see Fig 4 a4, b4, c4). Scatter plots in Fig 4 a5, b5, and c5 depict the individual P400 amplitudes, which are larger (i.e. above the bisection line) for disambiguated/high-visibility than for ambiguous/low-visibility stimuli for the vast majority of participants (Necker lattices and abstract figures: 18 out of 19 participants respectively; smileys: 19 out of 19 participants).

### Comparison of P200 and P400 ERP effects across stimulus types

**Fig 5:**
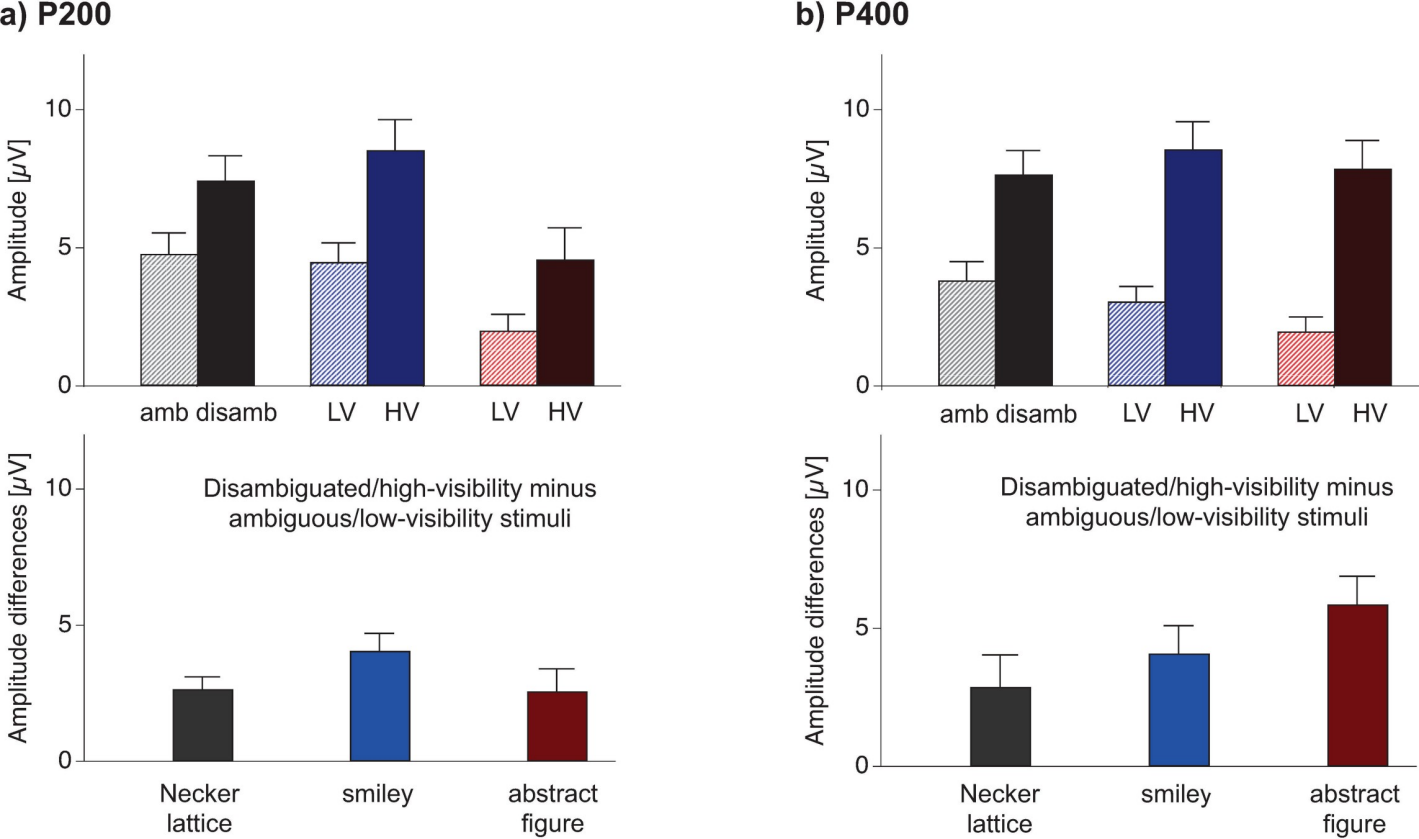
Grand mean P200 and P400 amplitudes. Top row: Grand mean amplitudes (±SEM) of the P200 (a) and P400 (b) ERP amplitudes are depicted for ambiguous (amb)/low-visibility (LV) and disambiguated (disamb)/high-visibility (HV) stimuli (upper row). Bottom row: Grand mean ERP amplitude differences for disambiguated/high-visibility minus ambiguous/low-visibility stimulus variants. All values result from the average around peak analysis (see methods section).

Fig 4 (a1), (b1), and (c1) depict similar P200 and P400 ERP Effects across stimulus types. Fig 5 upper row allows a direct comparison of the mean amplitudes (±SEM) for the P200 (a) and the P400 (b) ERP components.

Neither the rmANOVA on P200, nor on P400 ERP amplitude show a significant main effect for the fact *stimulus* (P200: *F*(2,36) = 3.85, *p* = 0.41; P400: *F*(2,36) = 0.81, *p* = 0.91). There were significant interactions between the factors *stimulus* and *sensory evidence* for both, the P200 (*F*(2,36) = 13.24, *p* = 0.001, $\eta_{p}^{2}$ = 0.42) and the P400 (*F*(2,36) = 40.97, *p* = 1e-08, $\eta_{p}^{2}$ = 0.69). In post-hoc *t-*tests we compared the peak differences between disambiguated/high-visibility and ambiguous/low-visibility stimulus variants between stimulus types (see Fig 5 bottom row). There were no significant effects of the peak differences between stimulus types in neither the P200 (Necker lattice vs. smiley: *t*(18) = -2.28, *p* = 0.42, Cohen’s d = 0.52; Necker lattice vs. abstract figure: *t*(18) = 0.09, *p* = 0.93, Cohen’s d = 0.02; smiley vs. abstract figure: *t*(18) = 1.91, *p* = 0.62, Cohen’s d = 0.44), nor in the P400 (Necker lattice vs. smiley: *t*(18) = -1.19, *p* = 0.92, Cohen’s d = 0.27; Necker lattice vs. abstract figure: *t*(18) = -2.84, *p* = 0.19, Cohen’s d = 0.65; smiley vs. abstract figure: *t*(18) = -2.7, *p* = 0.23, Cohen’s d = 0.62). We additionally calculated the effects size (Cohen’s d) of the difference between disambiguated/high-visibility and ambiguous/low-visibility conditions for each stimulus type separately. The results can be found in the following Table 2.

**Table 2:** Effect sizes ERP effects.

	P200	P400
Necker lattices	1.36	0.97
Smileys	1.44	2.13
Abstract figures	0.71	2.08

Table 2 displays effects sizes (Cohen’s d) for the difference between disambiguated/high-visibility and ambiguous/low-visibility conditions, separately for each stimulus type and ERP component (P200, P400).

In summary, we replicated the P200 and P400 ERP Effects for the Necker lattice stimuli [16,17] and found highly similar ERP Effects (concerning timing, location and amplitude effect sizes) for the smileys and abstract figures.

### N170 ERP results for smileys and abstract figures

The N170 is known to be related to face-specific processing [26] and can thus provide evidence for or against the processing of the smileys as faces. If smileys were perceived as faces, they should evoke N170 ERPs with larger (negative) amplitudes than the abstract figures. We tested whether this was the case in the present data.

**Fig 6:**
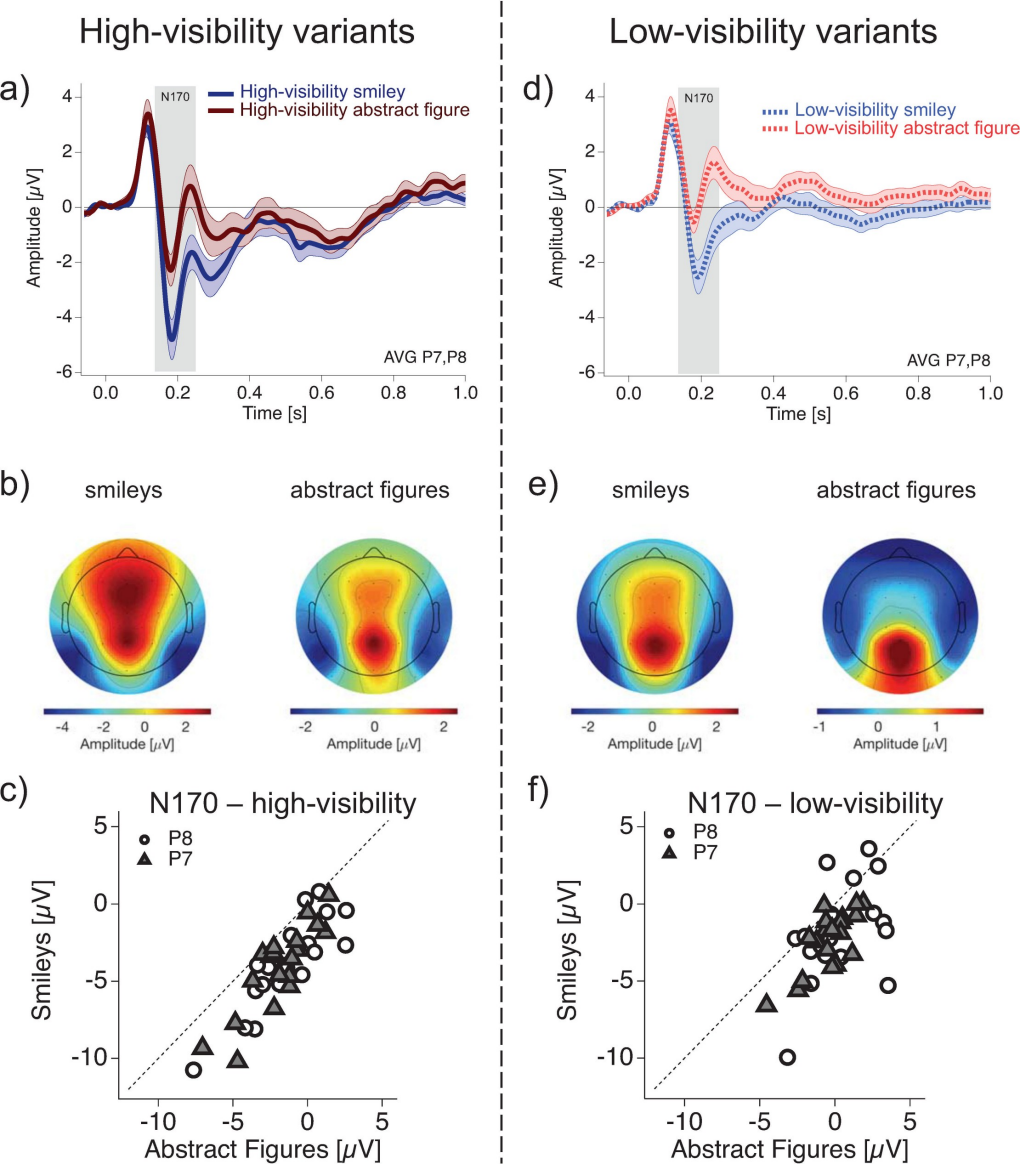
N170 for smileys and abstract figures. N170 effects (re-referenced to common average) in smileys and abstract figures. (a) and (d) depict the average of P7 and P8 grand mean data of smileys and abstract figures. (a) displays the ERP traces for the high-visibility variants (high-visibility smileys = dark blue solid line; high-visibility abstract figures = dark red solid line) and (d) for the low-visibility variants (low-visibility smileys = light blue dotted line; low-visibility abstract figure = light red dotted line). (b) and (e) show the respective grand mean scalp maps of the N170. The scatter plots in (c) and (f) show the N170 amplitudes from individual participants for smileys (ordinate) versus abstract figures (abscissa) for high-visibility (c) and low-visibility (f) stimulus variants at electrodes P7 (filled triangles) and P8 (hollow circles) separately. For both visibility levels, the vast majority of data points are below the bisection line indicating larger (more negative) N170 amplitudes for smileys than for abstract figures. No hemispheric difference is indicated.

Fig 6 displays the grand mean ERP data averaged across electrodes P7 and P8 and re-referenced to common average for the ambiguous/low-visibility (dotted lines, light colours) and disambiguated/high-visibility stimuli (solid lines, dark colours). Fig 6 (A) shows the ERP traces from high-visibility smileys (dark blue solid line) and high-visibility abstract figures (dark red solid line). Fig 6 (D) displays the grand mean data of low-visibility smileys (light blue dotted line) and low-visibility abstract figures (light red dotted line). The ERP traces in Fig (6A and 6D) show the same topology, with a positive deflection at around 150 ms, followed by a negative deflection at around 180 ms (the N170). The maximal (negative) excursion of the N170 is around electrodes P7 and P8 for high-visibility smileys, high-visibility abstract figures, and for low-visibility smileys (see voltage maps in Fig 6B (left and right) and e (left)), which is in accordance with the literature [26]. The N170 of the low-visibility abstract figures shows the smallest negative excursions, staying close to zero. This may explain the different voltage distribution across electrodes for this condition as displayed in the voltage maps (Fig 6E right).

The rmANOVA of the N170 ERP amplitude showed a significant main effect of *stimulus* (*F*(1,18) = 61.69, *p* = 9e-06, $\eta_{p}^{2}$ = 0.77). The amplitude differences between smileys and abstract figures can be seen in Fig 6 (C) for high-visibility and in Fig 6 (f) for low-visibility stimulus variants.

The rmANOVA further showed a significant main effect of *sensory evidence* (*F*(1,18) = 131.45, *p* = 3e-08, $\eta_{p}^{2}$ = 0.88), while no main effect for the factor *electrode* (*F*(1,18) = 1.32, *p* = 0.92) indicated no detectable hemispheric difference. No interactions were indicated between none of the factors (for details see Table C in Supporting Information S2 File).

In summary, we found larger (negative) N170 amplitudes for smileys compared to abstract figures. We further found larger (negative) N170 amplitudes for high-visibility compared to low-visibility stimulus variants.

## Discussion

The current study focused on two large ERP amplitude effects, labelled as the ERP Ambiguity Effects [16,17]: two ERP components (P200 and P400) show small amplitudes for ambiguous stimuli and large amplitudes for disambiguated stimulus variants. So far, these effects were found across very different lower (geometry, motion) and intermediate levels (Borings Old/Young Woman) of stimulus ambiguity. They have thus been attributed to stimulus ambiguity. In the present experiments, we investigated whether the ERP Ambiguity Effects can also be evoked by faces with high vs. low visibility of emotional expressions and in a control conditions with high and low visibility of low-level visual feature, namely the degree of curve bending.

We replicated these two ERP amplitude effects for Necker lattice stimuli and found similar effects for smiley faces and for abstract figures.

Perception of face stimuli is known to evoke larger N170 ERP components than non-face objects [26]. Larger (negative) amplitudes of the N170 ERP component for smileys compared to abstract figures thus indicate that the smileys were indeed processed as faces.

Median reaction times showed neither effects of sensory evidence (ambiguous/low-visibility, disambiguated/high-visibility) nor of stimulus type (Necker lattices, smileys, abstract figures). However, reaction times variability was overall larger for ambiguous/low-visibility than for disambiguated/high-visibility stimuli.

### Can we really compare ambiguity in Necker lattices, smileys and abstract figures?

The term "ambiguity" is often used in the sense that one and the same sensory information is compatible with more than one interpretation. In the case of the classical ambiguous figures, like the Necker cube, the sensory information is most compatible with two interpretations and perception oscillates between them. We do not have this binary situation with two distinct perceptual experiences when looking at the low-visibility smileys. However, the results of our psychophysical pilot study, reported in the Supporting Information S1 File show that the smileys with low visibility of the mouth curvature (close to the inflection point of the sigmoidal function in Fig A in Supporting Information S1 File) can be sometimes perceived as happy and sometimes as sad. Further, the high visibility stimulus variants are most often perceived in accordance with the intended respective stimulus manipulation. The stimuli from the two categories are thus located an about comparable perceptual scales.

It is of course possible to execute the task of the smiley pilot study as well as the task in the subsequent EEG study simply by focusing on the mouth curvature, while ignoring the face information from the smileys. One obvious question is thus, whether the smileys are indeed processed as faces. Overall larger P200 amplitudes for smileys than for abstract figures indicate principle differences in their processing even though the low-level features, i.e. luminance and overall line length were identical. Further, the larger N170 ERPs for smileys than for abstract figures provide physiological evidence for face-specific processing in the former case. A detailed analysis of the N170, including independent component analysis (ICA), can be found in the Supporting Information S3 File. Additionally, several studies indicate that our perceptual system automatically interprets any information with an approximately face-like structure as a face [36–38]. Taken together, these arguments make it very probable, that the smileys are perceived as faces.

Assuming face-perception mechanisms for the smileys, a second question is whether perceptual uncertainty in the case of smiley stimuli occurs at the level of face-emotion decoding, or at the level of the mouth curvature processing, or both? Differences in the overall pattern of the P200 and P400 ERP Effects between smileys and abstract figures would provide evidence for the former. This, however, would not stand in line with the generality of the ERP Effects found so far [16,17].

In fact, we did find the same overall pattern of P200 and P400 ERP Effects in Necker lattice, smiley, and abstract figure stimuli, even though effect sizes differ between stimulus types (for further discussion see below). This finding is in line with the overall pattern of generality of the ERP Effects across stimulus types, indicating processing differences at an abstract level beyond lower-level stimulus-specific processing steps (see discussion below). Our N170 interpretation strongly indicates that smileys are processed in a face-like manner. But based on the present data we cannot say whether the curvature is perceptually resolved during an early visual processing step, or alternatively during a holistic, higher-level face and emotion processing step. Similarly, we do not know whether the ambiguity resolution in the Necker lattice takes place at the level of single lines or at the level of bound line object (= lattice).

Numerous studies about classical ambiguous figures as well as studies in the context of the predictive coding theory discuss perception as a decision process based on probabilities [39–41]. The perceptual decision task is thus about finding the most probable interpretation for the given sensory information with a given visibility and/or ambiguity level. The probabilities of perceptual interpretations in turn are influenced by a number of factors, like previous perceptual experiences on different memory time scales. Studies about priming and adaptation both in the ambiguous figure literature [e.g. 42–46] but also in the face perception literature [e.g. 47,48], the emotional face expression literature [49] and beyond [50,51] indicate influence of the immediate perceptual history on the present percept. Studies about an a priori bias e.g. in the case of the Necker cube [52] but also the face inversion effect [53,54] indicate the importance of longer-term memory. Perceptual probability values can further be influenced by the current context [55], by observers intents and goals, but also by task instruction in experiments about perception in the lab [e.g. 56,57].

In the case of classical ambiguous figures, the underlying probability distribution is discrete (mostly bimodal, almost binary) in nature. In the case of the Necker cube, for example, 90° interpretations are most probable, because we live in a world with many 90° angles, although many other perceptual interpretations are principally possible [see Fig 2C in 15]. The task of the current paradigm, asking for a binary perceptual decision, is compatible with this. The "natural" probability distribution for the low-visibility smiley is most probably not binary. The strategy of the current study was to "binarize" the probability distribution of the low-visibility smileys and of the low-visibility abstract figures with our choice of stimuli and with the binary task.

### Ambiguity, probability, perceptual decision, and meta-perception–about the functional roles of the ERP effects

#### P200 and P400 in the literature

The currently investigated ERP Effects consist of amplitude differences in two ERP components, a centro-frontal P200 and a centro-parietal P400. The question is, what kind of neural processing do the P200 and the P400 amplitude effects reflect?

The results from the P200 time window in the current study indicate that there are two positivities at around 200 ms, one is a posterior P200, the other a more anterior P200 ERP component. The posterior P200 seems to be equally large as the anterior P200 for Necker lattice and smiley stimuli and even larger for the abstract figures (see voltage maps in Fig 4, right). The posterior P200 in the present data is most likely related to latest stages of sensory processing of the stimuli and probably related to posterior P200 components from the literature. In the literature, the posterior P200 is evoked by stimuli from different modalities [e.g. 58 for a visually evoked P200] although most studies used auditory stimuli [59]. Melloni et al. [60] modified the visibility of a letter embedded in varying levels of noise. They found a right-lateralized posterior P200, which was inversely related to letter visibility and depended on prior stimulus knowledge.

Only a few studies reported an anterior P200, similar to the present P200. Amongst these are Luck & Hillary [61], studying feature detection across visual dimensions, Taosheng et al. [62], studying modality-independent emotional salience, and Curran & Dien [63], studying the match of sensory input with memory contents [see also 64,65]. Kornmeier et al. [16,17] and a recent study [66] report a fronto-central P200 in the context of visual ambiguity (see also discussion below). These results about the anterior P200 provide evidence for its functional role beyond early sensory processing. In a recent study from our lab, we further found evidence for a functional separation between the P200, reflecting the ambiguity level of working memory information, and the P400, reflecting the integration of working memory content and sensory evidence [64].

The P400 is similar to the well-known P300 [specifically the P3b, see 67], which typically occurs in “oddball paradigms”: the P300 occurs between 250 ms and 600 ms after onset of an infrequent and task-relevant target stimulus (the “oddball”) or after infrequent omissions of a periodical stimulus. The P300 latency is negatively correlated with reaction times and its amplitude is negatively correlated with the target stimulus' frequency and positively correlated with stimulus discriminability [for recent reviews see 68,69]. One reason for our experimental block design, with either only ambiguous or only disambiguated stimuli, and for focusing only on stability trials and with this removing trials with oddball-like reversal events, was to avoid such an oddball P300 response. Delplanque et al. [70] investigated P300 ERPs evoked by oddball stimuli comprised of faces with unpleasant, pleasant or neutral emotional expressions. They found a typical P300 oddball effect with larger P300 amplitudes for faces with emotional valence compared to neutral faces. They assume a separate emotion-processing step on top of the oddball processing. The latter may be in line with our P400 findings in response to the smiley stimuli.

All of these arguments and observations indicate that the P400 cannot be reduced to the classical P300, although the two components may share some neural generators. In their former publication Kornmeier et al. [17] discuss this issue in more detail.

We recently found that the P200 and P400 ERP Effects are only present if the ambiguous and disambiguated stimuli are in the attentional focus [71,72]. This indicates that a certain relevance of the perceptual outcome, e.g. for the execution of a task, is a necessary precondition of the P200 and P400 ERP Effects. The processes underlying the ERP Effects are thus not executed automatically when the related sensory information is present. This is further evidence for higher-level processing steps related to attention and task-relevance.

**Can reversal rates explain the ERP amplitude effects?** The physical reversal rates for the disambiguated lattice stimuli as well as for low-visibility and high-visibility smileys and abstract figures were predefined to 30% by the stimulus program. This is about the rate of the typical reversal rates found for classical ambiguous figures, like the Necker cube [3,73]. As can be seen in Table 1 the perceptual reversal rates for the Necker lattice are in the expected range.

The perceptual reversal rates for the disambiguated lattice variants and the high-visibility smileys and abstract figures are in good confirmation with the predefined physical reversal rates. This result was expected because of the high visibility of the relevant curvature within the stimuli.

The perceptual reversal rates for the low-visibility smileys and abstract figures are roughly by factor 6 smaller than their predefined physical reversal rates of 30%. Given the low visibility of the curvature in these stimuli we expected a partial de-synchronization between physical and perceptual reversal events. However, the large decrease of reversal percepts translates into an increase of stability responses. This may indicate the influence of a priori perceptual biases (e.g. a preference for happy faces or upward bending), and/or perceptual priming evoked by the perception of immediately preceding stimuli. Further the large amount of stability responses may indicate that participants did not perceive the emotion/line bending as one of the two given options (e.g. smileys are perceived as neutral and neither as happy nor as sad) and a response strategy (e.g. preference for perceptual stability) would be the consequence. We did not collect information about individual strategies. Thus, we can neither analyse nor rule out the potential influence of such individual strategies to overcome low visibility and perceptual uncertainty on the ERP amplitude effects.

However, as already mentioned in the introduction there are qualitative differences between the perception of the ambiguous Necker lattice and the low-visibility smileys and abstract figures. At each moment we seem to have a clear and distinct 3D percept of the Necker lattice, which is one of the two most probable 90°-angle interpretations. The perceptual decision is thus an either-or decision. In the case of low-visibility smileys and abstract figures the perceptual decisions are rather a more-or-less decisions and the binarity only comes from the task constriction. This qualitative difference may be one reason for the quantitative difference in the perceptual reversal rates between the Necker lattices (close to 30%) and the low-visibility smileys and abstract figures (around 6%).

Given these and other differences it is even more remarkable that the ERP amplitude effects are that similar across stimulus types. The latter indicates that these effects reflect processes that are also beyond these differences, which we will discuss below.

**Do the P200 and P400 ERP Effects reflect stability of neural representations underlying percepts?** As already discussed above, because the sensory information is a priori incomplete, noisy and to varying degrees ambiguous, disambiguation, interpretation, and finally a perceptual decision become necessary. This decision is easy if a good quality of sensory information makes one interpretation highly probable compared to other theoretically possible interpretations. The resulting perceptual outcome will then be stable and reliable. In the case of ambiguity, and/or low visibility, perceptual decisions become more difficult and perceptual outcomes less stable over time, possibly resulting in spontaneous perceptual alternations as known from classical ambiguous figures.

It may be possible that the amplitude differences found for the P200 and P400 simply reflect differences in stability of neural representations. If this would be the case, we should expect that the neural representations of motion stimuli (e.g. the SAM), geometric cube stimuli (e.g. the Necker lattices) and face stimuli (e.g. the smileys) differ between each other, because they should be differently represented in the brain. However, the temporal and spatial patterns of our ERP Effects are surprisingly similar between these different stimulus categories. Further, the factor *stimulus* from the rmANOVA as well as the post-hoc t-tests did not indicate a significant difference of the amplitude effects between stimulus types.

Of course, it is not possible to make strong inferences from spatial distributions based on EEG voltage maps, and null-results from statistical tests do neither allow for far-reaching interpretations. Further, the significant interactions between the factors *stimulus* and *sensory evidence*, as indicated in the rmANOVAs, and differences in the sizes of the amplitude effects between stimulus categories (see Table 2) point to some differences. However, taking into account the large differences between stimulus categories (low-level features, but also conceptual differences), the identified ERP amplitudes are remarkably similar across stimulus categories.

EEG source analyses combined with fMRI data may resolve whether similarities on the scalp are based on the same underlying sources across stimuli. We are currently running an fMRI study investigating this question. Preliminary results indicate common sources across stimulus categories, making it less likely that the P200 and P400 ERP Effects reflect stability of perceptual / neural representations [74].

**Do the P200 and P400 ERP Effects reflect meta-perceptual processing?** An alternative interpretation of the present ERP amplitude effects is related to the recently discussed concept of meta-perception/ visual confidence. According to a definition given by Mamassian [41] meta-perception / visual confidence is "[…] the ability to estimate the accuracy of our visual decisions […]", and with this it is "[…] a judgment on a judgment […] ".

A closer look into the literature provides a rough time scale of visual processing and a very good guideline in this respect is the paper by Thorpe & Fabre-Thorpe [75]. Processing of objects and faces already takes place between 80 and 100 ms after stimulus onset and categorical judgements and decision making can be measured at about 120–160 ms after stimulus onset, as measured in monkeys. Values from humans may slightly differ and of course such values also strongly depend on stimulus identities. However, comparable values from humans indicate at least similar time scales [76–79]. Latencies of 200 ms (P200) and 400 ms (P400) thus indicate that the effects we found are most probably post-decision processes and may thus rather reflect estimations about the reliability of perceptual decisions.

We thus postulate that a secondary, meta-perceptual instance may evaluate the stability of neural/perceptual representations and the P200 and P400 amplitudes may reflect the evaluation result–or in other words–the certainty of our perceptual decision, with large amplitudes in the case of high reliability and vice versa. Assuming that this meta-perceptual instance is beyond sensory details—perhaps even beyond modalities—this approach may nicely explain the generality of our effects. In this case our results would present remarkably strong ERP correlates of meta-perceptual processing. It is important to add that meta-perceptual processing is not necessarily conscious processing. It is well possible and even probable that such meta-perceptual evaluations take place subconsciously in the majority of the cases and do not necessarily influence the quality of our conscious perceptual experience. Evaluations may only become conscious if their results involve substantial consequences for the current goals and the immediate behaviour.

## Conclusion and outlook

The present results further extend the generality of the P200 and P400 ERP Effects across stimulus types, with larger amplitudes for disambiguated/high-visibility compared to ambiguous/low-visibility stimulus variants. Importantly the effects were not only shown for classical ambiguous figures, but also for stimuli with low visibility of certain stimulus features, i.e. curvature. In future experiments it would be interesting to investigate the ERP Effects in other modalities like audition and touch.

We currently interpret the generality of the ERP Effects as an indication for meta-perceptual evaluations beyond sensory details and categories, and thus as an indication of certainty or uncertainty of a perceptual decision. As an important next step on our agenda to test this interpretation, the present paradigm will be extended by a confidence judgement as a second task. It would be further support of our hypothesis, if one and the same ambiguous stimulus were to elicit P200 and/or P400 amplitude modulations as a function of confidence ratings. Another step could be to compare ERP results from one experiment with classical ambiguous stimuli and disambiguated stimulus variants with ERP results from a second experiment where the unambiguous stimuli embedded in high and low visual noise will be compared. Visual noise is another–ecologically reasonable–way to modulate stimulus visibility. Preliminary results from our lab indicate highly similar P200 and P400 amplitude effects with noise stimuli [80].

Stating that both, the P200 and the P400 effects correlate with meta-perceptual processing is still rather unspecific. The difference in latency between P200 and P400 of 200 ms, which is quite long on perceptual processing time scales, indicates at least two separate processing steps. An interesting question is thus about the functional difference between P200 and P400. An important aspect of our paradigm is, that a current stimulus needs to be compared with the percept of the previous one. Thus, access to working memory content is necessary in order to execute this task. Recent results from our lab provide evidence that the working memory stores the identity of a previously perceived stimulus along with its ambiguity level and/or a corresponding reliability label. We found that the P200 effect can be explained almost entirely by such a working memory effect [65]. This finding is in good confirmation with theories about meta-perceptual rating and predictive coding approaches [81,82] emphasizing temporal aspects (i.e. memory and prediction) of perception and meta-perception.

The P400 latency and the present reaction times have similar latencies. Ambiguous sensory input results in unstable and thus unreliable percepts and uncertain motor decisions during task execution, i.e. which key to press. The P400 might reflect processes at the intersection of perception, meta-perception and motor execution. However, uncertainty during motor execution should result in more variable and overall longer reaction times compared to certain situations. In response to ambiguous sensory input we found more variance but no increase in reaction times compared to unambiguous input, which puts this interpretation into question. More studies are necessary to further clarify the mechanisms underlying the P200 and the P400 effects.

In conclusion, we here report ERP Effects with a large degree of generalization across different stimulus material. These effects show exceptionally large effect sizes and are clearly visible in almost all participants, which is rather uncommon for ERP effects, because of the known a priori low signal-to-noise ratio of EEG [83]. This may make these effects interesting in clinical contexts and related studies are currently in progress. In view of what we know so far about the ERP Ambiguity Effects we favour explanations in the context of meta-perceptual confidence judgments and (un)certainty of perceptual decisions. If this direction of interpretation will be confirmed in subsequent studies, we need to think about re-labelling this effect from "ERP Ambiguity Effect" to "ERP Uncertainty Effect" or "ERP Confidence Effect".

## Supporting information

S1 File Psychophysical pilot study to identify low-visibility smileys. (DOCX)

S2 File ERPs—statistical results. (DOCX)

S3 File Face perception and the N170 ERP component. (DOCX)

S1 Table Reaction times—statistical results. (DOCX)
