# Supplementary material for: Large EEG amplitude effects are highly similar across Necker cube, smiley, and abstract stimuli
Source: PLoS One. 2020 May 20;15(5):e0232928. doi: 10.1371/journal.pone.0232928 (PMC7239493; doi:10.1371/journal.pone.0232928)
Supplement: S1 File — (DOCX) [file pone.0232928.s001.docx]

**S1 File. Psychophysical Pilot Study to identify low-visibility Smileys.**

The present study used ambiguous and disambiguated variants of Necker lattices and low-visibility and high-visibility stimulus variants of smileys and abstract figures. The perceptually ambiguous and disambiguated lattice stimuli were adopted from previous studies [1,2]. We used smileys with low-visibility of their emotional expression, in contrast, for the first time in this study. It was necessary to access the perceptual scale of visibility of the emotional expressions as a function of the stimulus variable 'mouth curvature'.

We thus conducted a pilot study with 7 participants to determine probabilities of happy and sad face percepts as a function of the parameter mouth curvature using the method of constant stimuli [3]. All participants gave their informed written consent. The study was approved by the ethics committee of the University of Freiburg and in accordance with the ethical standards laid down in the Declaration of Helsinki [4].

10 smiley variants were presented in random order 10 times for 800 ms each (ISI = 200 ms) and participants were asked to indicate for each smiley stimulus whether it was perceived as happy or sad. The smiley variants differed in their mouth curvature with radii of r = 11.237° VA (variants 0 and 9), r = 14.422° VA (variants 1 and 8), r = 20.193° VA (variants 2 and 7), r = 33.527° VA (variants 3 and 6), and r = 100.662° VA (variants 4 and 5) (see Methods section).

Psychometric functions were fitted to each participant's average responses (across the ten stimulus repetitions) and averaged across participants. Figure A depicts the psychometric function of the averaged data across participants (±SEMs). The sigmoid inflection point indicates the most low-visibility smiley variant, i.e. where both, happy and sad smiley percepts are equally likely.

One problem with the so identified low-visibility smiley stimuli is that they are not perceived in a binary manner as the ambiguous lattices. In the case of ambiguous lattice stimuli, observers typically alternate spontaneously between two perceptual interpretations with precisely defined spatial angles. In contrast there are typically no spontaneous alternations between a clearly happy and a clearly sad percept if a smiley variant around the inflection point is presented (e.g. smiley 4 in Supporting Information Fig. S1). Rather the emotional expression of smiley 4 (the identified low-visibility variant) is perceived as slightly happier or sadder or even as neutral. To circumvent this problem, we took the two smiley variants closest to the inflection point (smiley 4 and smiley 5 as low-visibility smiley variants in the main study (see also the Discussion section for further elaboration on this aspect) and used a task with only two response options (perceptual stability and perceptual reversals). To ensure clear percepts, the high-visibility smileys shown in the main study had a stronger mouth bending than the smileys variants 0 and 9 from this pilot study.

For the control experiment in the main EEG study we followed the above described logic to decide for appropriate stimulus variants and simply isolated the mouth variants of these four smiley variants and embedded them to newly created abstract figures.

|  |
| --- |
| **Supporting Information File 1 - Fig A Pilot study to identify low-visibility mouth curvatures in smileys.** The top row depicts the psychometric function of the grand means (across participants) displaying the probability of perceived sadness as a function of the randomly presented smiley variants (± SEM). At the bottom six examples of smiley variants are depicted (from left to right: smiley variants 0, 2, 4, 5, 7, 9). Smiley variants 4 and 5 were used as low-visibility (of the mouth curvature) variants. High-visibility smiley variants, used in the main study had even sharper mouth curvatures than smileys 0 and 9 to ensure clear percepts. |

**References**

[1] Kornmeier J, Heinrich SP, Atmanspacher H, Bach M. The reversing “Necker Wall” – a new paradigm with reversal entrainment reveals an early EEG correlate. ARVO 2001 Annu. Meet., vol. 42, 2001, p. 409.

[2] Kornmeier J, Bach M. Early neural activity in Necker-cube reversal: Evidence for low-level processing of a gestalt phenomenon. Psychophysiology 2004;41:1–8. https://doi.org/doi: http://dx.doi.org/10.1016/j.visres.2004.10.006.

[3] Ehrenstein WH, Ehrenstein A. Psychophysical Methods. In: Windhorst U, Johansson H, editors. Mod. Tech. Neurosci. Res., Berlin, Heidelberg: Springer Berlin Heidelberg; 1999, p. 1211–41. https://doi.org/10.1007/978-3-642-58552-4_43.

[4] World Medical Association. Declaration of Helsinki: ethical principles for medical research involving human subjects. JAMA 2000;284:3043–5. http://dx.doi.org/10.1001/jama.284.23.3043.
