# Supplementary material for: Large EEG amplitude effects are highly similar across Necker cube, smiley, and abstract stimuli
Source: PLoS One. 2020 May 20;15(5):e0232928. doi: 10.1371/journal.pone.0232928 (PMC7239493; doi:10.1371/journal.pone.0232928)
Supplement: S2 File — (DOCX) [file pone.0232928.s002.docx]

**S3 File. ERPs - statistical results.**

**Supporting Information S3 File – Table A. ERP Effects - Repeated measures ANOVA on *ERP Amplitude* at electrode Cz.**

|  | Factor | F-Value | p-value corrected (uncorrected) |
| --- | --- | --- | --- |
| P200 | Stimulus | 3.85 | 0.41 (0.03) |
|  | Sensory evidence*** | 37.6 | 0.00023 (9e-06) |
|  | Stimulus : Sensory evidence** | 13.24 | 0.0011 (0.00005) |
|  |  |  |  |
| P400 | Stimulus | 0.81 | 0.91 (0.45) |
|  | Sensory evidence ** | 27.15 | 0.0013 (5e-05) |
|  | Stimulus : Sensory evidence*** | 40.97 | 1e-08 (5e-10) |

Repeated measures ANOVA results for the amplitudes of the two ERP components P200 and P400 at electrode Cz with Bonferroni-Holm corrected (and uncorrected p-values). The factor *stimulus* has three levels: Lattice, Smiley, Abstract Figure. The Factor *sensory evidence* has two levels: disambiguated/high-visibility and ambiguous/low-visibility (Significance Codes: p<0.05*; p<0.01**; p<0.001***).

**Supporting Information S3 File - Table B. Post-hoc t-tests for the interaction of *stimulus*sensory evidence*.**

|  | Comparison | t-value | p-value corrected (uncorrected) | Cohen’s d |
| --- | --- | --- | --- | --- |
| P200  (D-A) | Lattice vs. Smiley | -2.28 | 0.42 (0.04) | 0.52 |
|  | Smiley vs. Abstract Figure | 1.91 | 0.62 (0.07) | 0.44 |
|  | Lattice vs. Abstract Figure | 0.09 | 0.93 (0.93) | 0.02 |
|  |  |  |  |  |
| P400 (D-A) | Lattice vs. Smiley | -1.19 | 0.92 (0.25) | 0.27 |
|  | Smiley vs. Abstract Figure | -2.7 | 0.23 (0.01) | 0.62 |
|  | Lattice vs. Abstract Figure | -2.84 | 0.19 (0.01) | 0.65 |

Post-hoc t-tests for rmANOVA result of a significant interaction (*stimulus*sensory evidence*) at electrode Cz. Peak differences between disambiguated/high-visibility and ambiguous/low-visibility stimulus variants were calculated and p-values are Bonferroni-Holm corrected (and uncorrected p-values; Significance Codes: p<0.05*; p<0.01**; p<0.001***).

**Supporting Information Table S3 File – Table C.** **N170 - Repeated measures ANOVA on *ERP Amplitude.***

|  | Factor | F-value | p-value corrected (uncorrected) |
| --- | --- | --- | --- |
| N170 | Stimulus*** | 61.69 | 9e-06 (3e-07) |
|  | Sensory evidence *** | 131.45 | 3e-08 (1e-09) |
|  | Electrode | 1.32 | 0.92 (0.27) |
|  | Stimulus : Sensory evidence | 0.51 | 0.74 (0.49) |
|  | Stimulus : Electrode | 0.23 | 0.64 (0.64) |
|  | Sensory evidence: Electrode | 0.46 | 0.51 (0.51) |
|  | Stimulus : Sensory evidence : Electrode | 0.02 | 0.89 (0.89) |

Repeated measures ANOVA results for the amplitudes of the N170 ERP component with Bonferroni-Holm corrected p-values (uncorrected p-values). The factor *stimulus* has two levels: Smiley, Abstract Figure. The factor *sensory evidence* has two levels: disambiguated/high-visibility and ambiguous/low-visibility. The factor *electrode* has two factors: P7 and P8 (Significance Codes: p<0.05*; p<0.01**; p<0.001***).
