# Supplementary material for: Large EEG amplitude effects are highly similar across Necker cube, smiley, and abstract stimuli
Source: PLoS One. 2020 May 20;15(5):e0232928. doi: 10.1371/journal.pone.0232928 (PMC7239493; doi:10.1371/journal.pone.0232928)
Supplement: S3 File — (DOCX) [file pone.0232928.s003.docx]

**S4 File. Face Perception and the N170 ERP Component.**

Background

One of the major foci of the present experiments was to study ERP correlates of visibility of the emotional content of smiley face stimuli. We manipulated emotion by varying the smileys' mouth curvatures. One problem of this approach is that participants could simply distinguish between upward and downward oriented (mouth) curvatures to execute the task. Face perception is in principle not necessary.

To clarify whether the smileys were really perceived as faces, we presented the same mouth curvatures in the context of abstract figures as a control condition.

Face perception is known to evoke larger N170 ERP components than objects [5]. If smileys were perceived as faces, they should evoke N170 ERPs with larger amplitudes than the abstract figures.

Analysis overview

The Independent Component Analysis (ICA) was applied to isolate an independent EEG component that most probably represents the N170. The amplitudes of the respective components from smiley and abstract figure stimuli were then compared with each other. The basic steps were the following:

- ICA on each individual dataset
- Isolation of independent components (ICs) most likely representing the N170 ERP component
- Back-projection of the identified components into the electrode domain.
- Determination of how much of the originally found N170 ERP components can be explained by those components.

Analysis details

(A) Calculating ICA

For each participant and visibility level (low-visibility and high-visibility) the following pre-processing steps were realized:

- Pooling smiley and abstract figure data
- Down-sampling data to 250 Hz
- Selecting data from 60 ms before to 1000 ms after stimulus onset (only stability trials; see Methods for their definition)
- Rejecting artefacts (± 100 µV threshold)
- Concatenating single trials

On this pre-processed data an AMICA [6] was conducted using the implementation provided by the Matlab toolbox EEGLAB [7].

In the ICA the number of electrodes (here 32) determines the maximal number of identifiable independent components (ICs). The difference between low-visibility and high-visibility stimuli is prominent in the data and importantly, detected by the ICA. To not "loose" available ICs to the ERP difference related to stimulus visibility, but to extract the N170 related ICs, we decided to calculate separate ICAs for low-visibility and high-visibility stimuli.

(B) Selection of ICs related to the N170

The ICA provided 32 ICs per participant. The ICs that most likely contributed to the N170 component were selected as follows:

(B1) Rejection of ICs representing eye artefacts: ICs related to eye artefacts were identified with a cluster analysis based on dipole locations of the ICs close to the eyes and artefact typical activations [8]. Those ICs were excluded.

(B2) Selection steps for N170-related ICs:

- Selection of ICs with maximal activity located in the posterior hemisphere (TP9, CP5, CP1, CP2, CP6, TP10, P7, P3, Pz, P4, P8, PO9, O1, Oz, O2, PO10)
- Back-projection of the selected ICs into the electrode domain
- Selection of those ICs whose back-projection explained at least 5 % of artefact-free data (sum of the squared amplitude values) at the N170-specific electrodes P7 and P8 (spatial ROI) in a time-window from 170 to 220 ms (temporal ROI)

Results

Between 2 and 7 (median = 4) ICs per participant were found for the low-visibility and for the high-visibility stimuli related to the N170 ERP component. The sum of those ICs explained on average 61.5 % (median, range = 35.6-80.2 %) for the low-visibility stimuli and 72.5 % (median, range = 56.3-83.3 %) for the high-visibility stimuli of the originally found N170 amplitudes related to smileys and abstract figures.

As can be seen in the graphical illustration of the results (Fig A a-c and f-h), the modulation of the N170 (smileys vs. abstract figures) is almost identical between the original ERPs and the back-projected N170 related independent components. Importantly, the effect of larger N170 amplitudes of smileys compared to abstract figures is conserved in the back-projected N170 components from the IC analysis.

Discussion:

The P200 ERP and the N170 are in relatively close temporal vicinity. It is thus theoretically possible that the modulation of one of those ERP components as a function of stimulus type and / or visibility level may be also reflected in the other component due to volume conduction.

We applied the ICA in order to disentangle the two components and therewith isolate ERP specific effects. In particular we were interested in whether the N170 component shows face specific modulations with the smiley stimuli. We further aimed to check whether such a modulation is really related to the N170 or alternatively influenced by the P200 modulation.

We identified N170-specific independent components that show – after back-projection - the same face specific modulation as the originally identified N170. We regard this as evidence for face-specific processing of the smiley stimuli.

| **** |
| --- |
| **Supporting Information S4 File Fig B. Back projected ICA components - N170 Smiley vs. Abstract Figure.** Back projected N170 independent components in smileys and abstract figures, analysed separately for high-visibility and low-visibility stimuli. (a) depicts the grand mean back-projected ERP traces averaged across electrode P7 and P8 of smileys (blue) and abstract figures (red) for the high-visibility (solid lines) stimulus variants and (f) for the low-visibility (dotted lines) stimuli. Sub graphs (b, c, g, h) show the back-projected IC ERPs together with the original ERPs. The grand mean data was averaged across electrodes P7 and P8. Sub graphs (d) and (i) show the grand mean scalp maps of the N170. Sub graph (e) and (j) depict scatterplots of the N170 peak amplitudes from the back-projected data of individual participants by plotting smileys (ordinate) versus abstract figures (abscissa) for electrodes P7 (filled triangles) and P8 (hollow circles) separately. The vast majority of data points are below the bisection line, showing larger N170 amplitudes for smileys than for abstract figures. This indicates face-specific processing of the smileys. |

**References**

[1] Rossion B, Jacques C. The N170: Understanding the Time Course of Face Perception in the Human Brain. In: Kappenman ES, Luck SJ, editors. Oxf. Handb. Event-Relat. Potential Compon., Oxford University Press; 2011.

[2] Palmer JA, Kreutz-Delgado K, Makeig S. AMICA: An Adaptive Mixture of Independent Component Analyzers with Shared Components 2011:15.

[3] Delorme A, Makeig S. EEGLAB: an open source toolbox for analysis of single-trial EEG dynamics including independent component analysis. J Neurosci Methods 2004;134:9–21. https://doi.org/10.1016/j.jneumeth.2003.10.009.
