# Supplementary material for: Large EEG amplitude effects are highly similar across Necker cube, smiley, and abstract stimuli
Source: PLoS One. 2020 May 20;15(5):e0232928. doi: 10.1371/journal.pone.0232928 (PMC7239493; doi:10.1371/journal.pone.0232928)
Supplement: S1 Table — (DOCX) [file pone.0232928.s004.docx]

**S2 Table. Reaction times - statistical results.**

**Supporting Information Table S2. Reaction Time data - Wilcoxon tests**

| Dependent variable | Contrast | *Z* score (based on positive ranks) | Effect size *r* | *p*-value corrected (uncorrected) |
| --- | --- | --- | --- | --- |
| Median | **Sensory evidence (ambiguous/low-visibility vs. disambiguated/high-visibility)** | | | |
|  | Lattice | 1.05 | 0.17 | 0.93 (0.31) |
|  | Smiley | 0.54 | 0.09 | 0.58 (0.58) |
|  | Abstract Figure | 0.85 | 0.14 | 0.93 (0.42) |
|  | **Stimulus type (collapsed across ambiguity/visibility levels)** | | | |
|  | Lattice vs. Smiley | 1.01 | 0.16 | 0.91 (0.33) |
|  | Lattice vs. Abstract Figure | 1.65 | 0.27 | 0.72 (0.1) |
|  | Smiley vs. Abstract Figure | 0.77 | 0.12 | 0.85 (0.47) |
| Inter-quartile Range | **Sensory evidence (ambiguous/low-visibility vs. disambiguated/high-visibility)** | | | |
|  | Lattice*** | 3.78 | 0.61 | 0.0002 (7e-06) |
|  | Smiley** | 3.7 | 0.6 | 0.0005 (2e-05) |
|  | Abstract Figure** | 3.54 | 0.57 | 0.002 (7e-05) |
|  | **Stimulus type (disambiguated/high-visibility stimuli)** | | | |
|  | Lattice vs. Smiley** | 3.3 | 0.54 | 0.007 (0.0003) |
|  | Lattice vs. Abstract Figure | 2.05 | 0.33 | 0.44 (0.04) |
|  | Smiley vs. Abstract Figure | 2.13 | 0.35 | 0.41 (0.03) |
|  | **Stimulus type (ambiguous/low-visibility stimuli)** | | | |
|  | Lattice vs. Smiley | 1.25 | 0.2 | 0.93 (0.23) |
|  | Lattice vs. Abstract Figure | 0.32 | 0.05 | 0.77 (0.77) |
|  | Smiley vs. Abstract Figure | 1.53 | 0.25 | 0.78 (0.13) |

Bonferroni-Holm corrected (uncorrected p-values; Significance Codes: p<0.05*; p<0.01**; p<0.001***).
